# Transmission Dynamics by Age Group in COVID-19 Hotspot Counties — United States, April–September 2020

**DOI:** 10.15585/mmwr.mm6941e1

**Published:** 2020-10-16

**Authors:** Alexandra M. Oster, Elise Caruso, Jourdan DeVies, Kathleen P. Hartnett, Tegan K. Boehmer

**Affiliations:** 1 CDC COVID-19 Response Team.

CDC works with other federal agencies to identify counties with increasing coronavirus disease 2019 (COVID-19) incidence (hotspots) and offers support to state, tribal, local, and territorial health departments to limit the spread of SARS-CoV-2, the virus that causes COVID-19 (*1*). Understanding whether increasing incidence in hotspot counties is predominantly occurring in specific age groups is important for identifying opportunities to prevent or reduce transmission. The percentage of positive SARS-CoV-2 reverse transcription–polymerase chain reaction (RT-PCR) test results (percent positivity) is an important indicator of community transmission.* CDC analyzed temporal trends in percent positivity by age group in COVID-19 hotspot counties before and after their identification as hotspots. Among 767 hotspot counties identified during June and July 2020, early increases in the percent positivity among persons aged ≤24 years were followed by several weeks of increasing percent positivity in persons aged ≥25 years. Addressing transmission among young adults is an urgent public health priority.

Hotspot counties were identified by applying previously described standardized criteria to detect counties that had >100 cases during the past 7 days and experienced increases in cases in the preceding 3–7 days (*1*). Counties identified as hotspots during June 1–July 31, 2020, that had not met hotspot criteria in the previous 21 days were included. SARS-CoV-2 RT-PCR test results were obtained from data submitted by state health departments and laboratories.^†^ Percent positivity was calculated by dividing the number of positive test results by the sum of positive and negative test results for each age group (0–17, 18–24, 25–44, 45–64, and ≥65 years) for the 45 days before and 45 days after hotspot detection (spanning April–September 2020) based on specimen collection or test order date. Data were presented using a 7-day moving average. Results were aggregated across all hotspot counties and stratified by age group. Analyses were conducted using R software (version 3.6.0; The R Foundation).

The 767 hotspot counties detected during June 1–July 31 represented 24% of all U.S. counties and 63% of the U.S. population. Percent positivity among persons aged 0–17 and 18–24 years began increasing 31 days before hotspot identification. Increases in percent positivity among older age groups began after the increases in younger age groups: among adults aged 25–44 years, 45–64 years, and ≥65 years, increases began 28 days, 23 days, and 20 days, respectively, before hotspot identification (Figure 1). At the time of hotspot detection, the highest percent positivity was among persons aged 18–24 years (14%), followed by those aged 0–17 years (11%), 25–44 years (10%), 45–64 years (8%), and ≥65 years (6%). Percent positivity among persons aged 18–24 years was near its peak of 15% by the date of hotspot detection; however, among other age groups, percent positivity continued to increase for 21–33 days after hotspot detection, peaking at 10%–14%, and the decline for other age groups was slower than that for persons aged 18–24 years.

**FIGURE 1:**
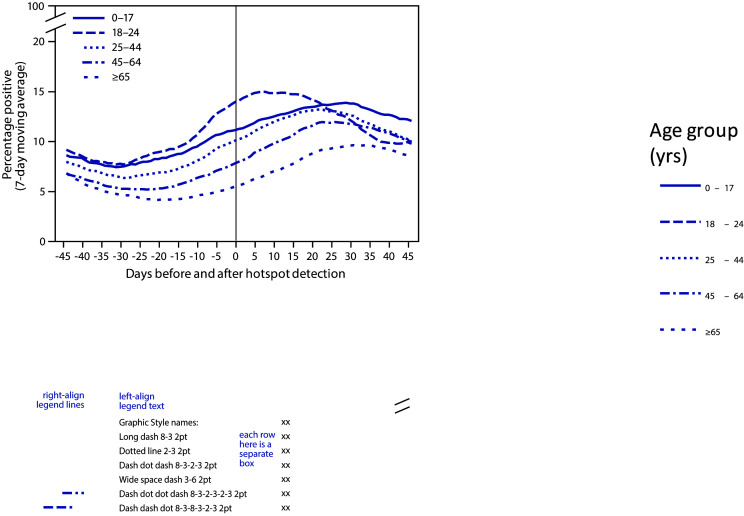
Percentage of positive SARS-CoV-2 reverse transcription–polymerase chain reaction test results (7-day moving average)* in COVID-19 hotspot counties before and after date of hotspot detection, by age group — United States, June 1–July 31, 2020 **Abbreviation:** COVID-19 = coronavirus disease 2019. * From COVID-19 electronic laboratory reporting data submitted by state health departments for 37 states and from data submitted directly by public health, commercial, and reference laboratories for 13 states and the District of Columbia, using specimen collection or test order date.

Important differences were identified when analyzing percent positivity by U.S. Census region^§^ (Figure 2). Trends by age for hotspot counties in the South (488 counties) and West (98 counties) aligned with national trends, although percent positivity was higher in the South than in the West for all age groups. In hotspot counties in the Midwest (134 counties), percent positivity among persons aged 18–24 years peaked before hotspot detection, and percent positivity increased minimally in other age groups. In hotspot counties in the Northeast (47 counties), there was a small increase in percent positivity among persons aged 18–24 years but minimal or no increases in other age groups.

**FIGURE 2:**
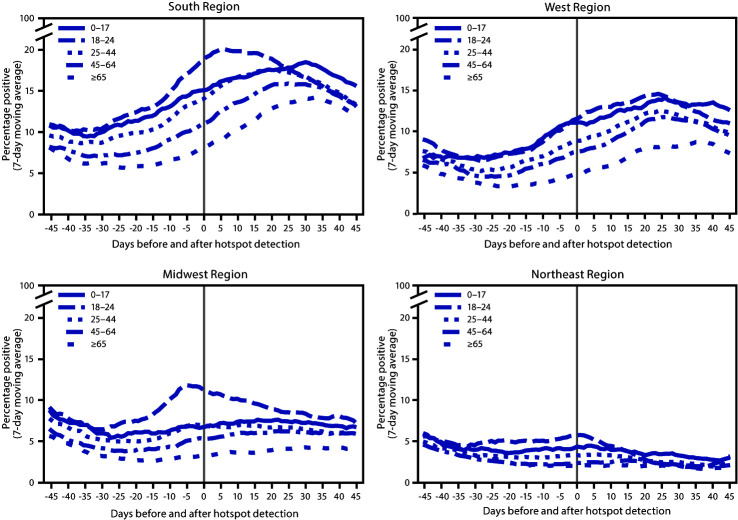
Percentage of positive SARS-CoV-2 reverse transcription–polymerase chain reaction test results (7-day moving average)* in COVID-19 hotspot counties before and after date of hotspot detection, by age group and U.S. Census region^†^ — United States, June 1–July 31, 2020 **Abbreviation:** COVID-19 = coronavirus disease 2019. * From COVID-19 electronic laboratory reporting data submitted by state health departments for 37 states and from data submitted directly by public health, commercial, and reference laboratories for 13 states and the District of Columbia, using specimen collection or test order date. ^†^ *South:* Alabama, Arkansas, Delaware, District of Columbia, Florida, Georgia, Kentucky, Louisiana, Maryland, Mississippi, North Carolina, Oklahoma, South Carolina, Tennessee, Texas, Virginia, and West Virginia. *West:* Alaska, Arizona, California, Colorado, Hawaii, Idaho, Montana, Nevada, New Mexico, Oregon, Utah, Washington, and Wyoming. *Midwest*: Illinois, Indiana, Iowa, Kansas, Michigan, Minnesota, Missouri, Nebraska, North Dakota, Ohio, South Dakota, and Wisconsin. *Northeast:* Connecticut, Maine, Massachusetts, New Hampshire, New Jersey, New York, Pennsylvania, Rhode Island, and Vermont.

In hotspot counties, particularly those in the South and West, percent positivity increased earliest in younger persons, followed by several weeks of increasing percent positivity among older age groups. An increase in the percentage of positive test results in older age groups is likely to result in more hospitalizations, severe illnesses, and deaths.^¶^ These findings corroborate regional patterns in the southern United States, where increased percent positivity among adults aged 20–39 years preceded increases among those aged ≥60 years (*2*); provide evidence that among young adults, those aged 18–24 years demonstrate the earliest increases in percent positivity; and underscore the importance of reducing transmission from younger populations to those at highest risk for severe illness or death. There is an urgent need to address transmission among young adult populations, especially given recent increases in COVID-19 incidence among young adults (*3*). These data also demonstrate the urgency of health care preparedness in hotspot counties,** which are likely to experience increases in COVID-19 cases and hospitalizations among older populations in the weeks after meeting hotspot criteria.

## Footnotes

* https://www.cdc.gov/coronavirus/2019-ncov/lab/resources/calculating-percent-positivity.html; https://www.whitehouse.gov/openingamerica/.

† SARS-CoV-2 RT-PCR testing data were obtained from COVID-19 electronic laboratory reporting data submitted by state health departments for 37 states and from data submitted directly by a subset of public health, commercial, and reference laboratories (representing approximately 50% of all tests) for 13 states and the District of Columbia. The data might not include results from all testing sites within a jurisdiction (e.g., point-of-care test sites) and therefore reflect the majority, but not all, SARS-CoV-2 RT-PCR tests in the United States. The data represent laboratory test totals (not individual persons) and exclude antibody and antigen tests.

§ https://www2.census.gov/geo/pdfs/maps-data/maps/reference/us_regdiv.pdf.

¶ https://www.cdc.gov/coronavirus/2019-ncov/need-extra-precautions/older-adults.html.

** CDC and other federal agencies that monitor trends in COVID-19 are collaborating to refine approaches to define and track hotspots. As a result, terminology or definitions used in future reports might differ from the terminology used in this report.
